# Spanish Questionnaires for the Assessment of Pelvic Floor Dysfunctions in Women: A Systematic Review of the Structural Characteristics and Psychometric Properties

**DOI:** 10.3390/ijerph182312858

**Published:** 2021-12-06

**Authors:** Marina Guallar-Bouloc, Paloma Gómez-Bueno, Manuel Gonzalez-Sanchez, Guadalupe Molina-Torres, Rafael Lomas-Vega, Alejandro Galán-Mercant

**Affiliations:** 1 Department of Physiotherapy, Health Science Faculty, University of Jaén, 23071 Jaén, Spain; marina.guallar@gmail.com (M.G.-B.); rlomas@ujaen.es (R.L.-V.); 2 Move-It Research Group, Department of Physical Education, Faculty of Education, Sciences University of Cádiz, 11002 Cádiz, Spain; paloma.gomezbueno@alum.uca.es (P.G.-B.); alejandro.galan@uca.es (A.G.-M.); 3 Department of Physiotherapy, Faculty of Health Sciences, University of Málaga, 29071 Málaga, Spain; mgsa23@uma.es; 4 Institute of Biomedicine of Málaga (IBIMA), 29010 Málaga, Spain; 5 Department of Nursing, Physiotherapy and Medicine, Faculty of Health Sciences, University of Almería, 04120 Almería, Spain; 6 Biomedical Research Unit, Innovation Institute of Cádiz (INiBICA), Puerta del Mar University Hospital, University of Cádiz, 11002 Cádiz, Spain

**Keywords:** pelvic floor dysfunctions, sexual dysfunction, urinary incontinence, prolapse, validation, questionnaire, Spanish

## Abstract

Background: Pelvic floor dysfunctions affect a third of the adult female population, including a large number of clinical conditions, which can be evaluated through validated questionnaires that inform us of the status and perception of women both objectively and subjectively. The main objective of this study was to review and explain the topics of the validated questionnaires in Spanish on pelvic floor dysfunctions and to review their psychometric properties. Methods: A systematic review was carried out in the PUBMED and WOS databases. The keywords used were in PUBMED: ((((((((“Fecal Incontinence” [Mesh]) OR “Urinary Incontinence” [Mesh]) OR “Pelvic Organ Prolapse” [Mesh]) OR “Pelvic Floor Disorders” [Mesh]) OR “Sexual Dysfunction, Physiological” [Mesh]) OR “Pelvic Girdle Pain” [Mesh]) OR “sexual function” [Title/Abstract]) OR “Prolapse” [Title/Abstract]) AND “Surveys and Questionnaires” [Mesh] AND “Validation” [Title/Abstract] combined with the Boolean operators “AND”/“OR”. In contrast, in WOS, a segregated search was carried out with each of the terms of pelvic floor dysfunction together with “Validation” and “Surveys and Questionnaires”. All articles published up to 19 November 2021 were considered. Methodological quality was assessed with the COSMIN scale. Results: A total of 687 articles were identified, of which 13 were included. The evaluated questionnaires and the structural characteristics and psychometric properties of each of them were collected. Conclusion: The Spanish versions of the questionnaires show good basic structural and psychometric characteristics for the evaluation of patients with pelvic floor dysfunctions and that they resemble other versions of the same questionnaire published in other languages.

## 1. Introduction

Pelvic floor dysfunction (PFD) is defined by Grimes and Stratton [1] and refers to a wide range of symptoms, signs and anatomical changes related to the abnormal function of the pelvic floor musculature and the structures with which it works synergistically. The pelvic floor is subjected to high pressures; its ability to resist them is due to the tonic-static activity of the aponeurotic structures and muscles that form it, but if these structures are weakened, hypotonic, or hypertonic, they are not able to readjust intra-abdominal pressure (IAP) [2], poor proprioception or histological problems, and different pathologies can occur that we can include in the concept of “pelvic floor dysfunction” [3,4,5]. PFD affects one third of the adult female population [6] and includes a large number of clinical conditions, such as urinary incontinence (UI), fecal incontinence (FI), pelvic organ prolapse (POP), alterations in perception or filling of the lower urinary tract, defecatory disorders (DD), sexual dysfunction and numerous chronic pain syndromes in the perineal area. This wide variety of problems affects women 3 to 7 times more than men [7]. Pelvic floor problems do not pose any risk to the lives of our patients, but we must be very aware of what they cause. This pathology influences different aspects, such as social, physical, psychological, occupational or sexual function, notably affecting the Quality of Life (QoL) of women who suffer from them [8].

Since Engel [9] postulated the need for a holistic medical model, which he called biopsychosocial, in response to the biomedical model, patient care has evolved towards a patient-centered clinical practice [10]. This proposal was well received by the sectors that wanted the incorporation of empathy and compassion in clinical practice. The biopsychosocial model is committed to giving the patient a say in the care process, going from being a mere object to being the subject of the clinical act [11]. Following this premise, the questionnaires provide us with information about our patient’s self-perception and facilitate the clinician’s access to this information. Having these data is essential to be able to understand the evolution one is experiencing. The use of patient-reported outcome measures (PROM) that ask patients to assess elements of their own health, QoL and functionality is also interesting. These data can provide information about how an intervention and treatment affect these aspects of a patient’s life [12].

For the evaluation of PFD, PFD assessment can be used, but it may vary depending on the healthcare provider that evaluates it. Additionally, another point to keep in mind is that there are patients who are not comfortable talking about this topic and are reluctant to ask clinical staff about their PFD [13,14]. These are two of the reasons why it is important to have questionnaires, for example PFDI-20 [8], PFIQ [8], IFSF [15], among others, that can be completed autonomously and easily, since they are written so that they are easily understood by patients [16].

Currently, the existence of tools such as validated questionnaires has become extremely important, since they allow us to objectively and subjectively evaluate the different symptoms of pathologies that can be found in the day-to-day clinical practice based on evidence [17]. The function of these questionnaires is mainly to facilitate objectively any response or variation in the state of health according to the perception of the patient. Thanks to them, clinicians can make a more complete diagnosis and carry out better management of the symptoms in question. Many of these questionnaires are used today as a standard protocol for measuring and managing a patient’s condition. All these measurement tools that are used in both clinical practice and in research must first undergo a validation process in which their psychometric characteristics are taken into account, as well as a validation of the language and culture of the each clinical population group.

According to the latest update of the Cervantes Institute [18], more than 585 million people speak Spanish worldwide, where almost 489 million are native Spanish speakers. Spanish is also the second mother tongue of number of speakers and the third most-spoken language globally after English and Mandarin Chinese [18]. Hence, the importance of conducting a study that groups and classifies all the psychometric questionnaires that evaluate the PFD existing up to now and that, in addition, are translated into Spanish to facilitate and simplify the clinical practice of a very large community such as Spanish speakers is clear.

The main objective of this study was to systematically review the existing scientific literature on cross-cultural adapted and validated questionnaires into the Spanish language of Spain for the evaluation of the main dysfunctions of the pelvic floor. As a secondary objective, the psychometric properties of the questionnaires that are located in the main objective of this work will be reviewed.

## 2. Material and Method

### 2.1. Protocol

A systematic review of the literature was carried out following the general guidelines and recommendations of the PRISMA statement [19] and was registered in the PROSPERO database (CRD no.: 42021279944).

### 2.2. Sources and Search

For the development of this systematic review, a systematic search was carried out in the PUBMED and WOS databases. The keywords used combined with the Boolean operators “AND”/“OR” were: (“Fecal Incontinence” [Mesh]) OR “Urinary Incontinence” [Mesh]) OR “Pelvic Organ Prolapse” [Mesh]) OR “Pelvic Floor Disorders” [Mesh]) OR “Sexual Dysfunction, Physiological” [Mesh]) OR “Pelvic Girdle Pain” [Mesh]) OR “sexual function” [Title/Abstract]) OR “Prolapse” [Title/Abstract]) AND “Surveys and Questionnaires” [Mesh] AND “Validation” [Title/Abstract]. In contrast, in WOS, a segregated search was carried out with each of the terms of pelvic floor dysfunction together with “Validation” and “Surveys and Questionnaires”. Articles published up to 19 November 2021 were considered.

### 2.3. Selection Criteria

The inclusion criteria used in this review were: cross-cultural adaptation and validation studies into Spanish (Spain) of questionnaires for the evaluation of the pelvic floor. All articles that were published in a language other than Spanish or English were excluded, and studies conducted only in women were considered. In addition, the methodological quality of the studies was evaluated following the criteria of the Consensus-Based Standards for the Selection of Health Measurement Instruments (COSMIN) tool [20]. The COSMIN scale develops a set of relevant measurement properties to assess patient-reported outcome measures (PROM) [21], which considers four domains: validity, reliability, responsiveness and feasibility, with the related measurement properties and their characteristics. For this study, it was decided to evaluate the domains of reliability (internal consistency and reliability), validity and responsiveness.

### 2.4. Selection of Documents

The bibliographic citations identified were transferred to a tool to collect, examine and evaluate the titles and summaries of the citations; this was the Rayyan platform (rayyan.qcri.org) [22]. In the first place, the duplicate articles found were eliminated, leaving a total of 13 documents.

Subsequently, two investigators (PGB-AGM) reviewed and screened independently and blinded by title and abstract, and articles that did not meet the inclusion criteria were eliminated. Those that, on the contrary, if they complied, were selected and located for their full-text reading, articles that were in doubt or when the title and abstract did not reveal enough information to determine their inclusion or exclusion were also retrieved. Discrepancies were resolved by a third reviewer (GMT).

### 2.5. Results Synthesis and Data Extraction

All the articles that were finally selected were analyzed and processed in order to identify validated questionnaires and gather information on the construction and validation process of these tools. The data extracted from each instrument were: full name, acronym, version, number of items, purpose of the questionnaire, scale, subscale, time to complete, scoring scale. The psychometric aspects extracted were: test–retest reliability, internal consistency, construct validity, content validity and standard error of measurement.

## 3. Results

**Figure 1:**
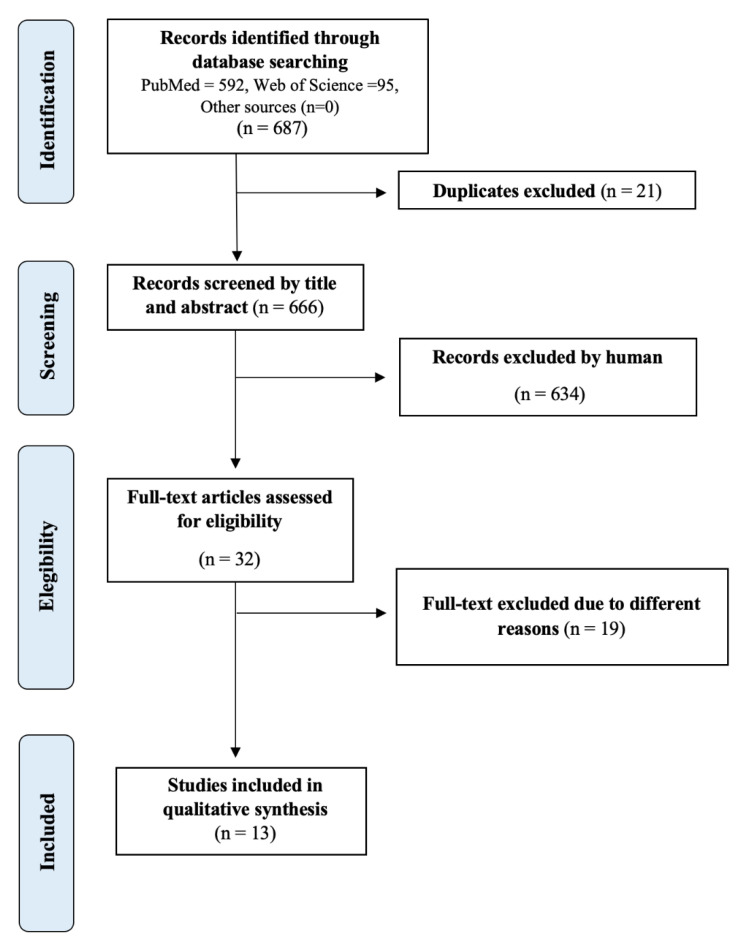
Instrument selection (flow chart).

After identification of 687 documents (PubMed = 592) and WOS (n = 95) and elimination of 21 duplicates, 666 documents were selected. Of this number of articles, 634 were finally excluded by reading the title and abstract, 18 for not being adaptations and validations of questionnaires or studying mixed or male population. Finally, we were left with a total of 13 articles to carry out our systematic review, reflecting this entire selection process in the flow diagram below (Figure 1).

After reading the titles and applying the selection criteria to the complete documents, 13 articles and a total of 15 questionnaires were selected [8,14,15,23,24,25,26,27,28,29,30,31,32].

These included four questionnaires that assess the QoL in people with PFD [15,26]: Urogenital Distress Inventory (UDI-6), Incontinence Impact Questionnaire (IIQ-7), Pelvic Floor Distress Inventory Short Form (PFDI-20) and Pelvic Floor Impact Questionnaire Short Form (PFIQ-7).

Four questionnaires assess the impact of PFD on a woman’s sexual life [15,24,25,31]: Pelvic Organ Prolapse/Urinary Incontinence Sexual Questionnaire (PISQ-12), Female Sexual Function Index (FSFI), Sexual Satisfaction Scale for Women (SSS-W-E), Pelvic Organ Prolapse/Urinary Incontinence Sexual Questionnaire IUGA- Revised (PISQ-IR), and Female Sexual Function Index in postmenopausal women (FSFI).

These questionnaires assess the symptoms of POP and UI as well as their classification and severity and how they affect women [14,23,24,30,31]: Incontinence Questionnaire-Short Form (ICIQ-SF), Bladder control Self-Assessment Questionnaire (B-SAQ), Prolapse and Incontinence Knowledge Questionnaire (PIKQ), Epidemiology of Prolapse and Incontinence Questionnaire (EPIQ) and Prolapse Quality of Life Questionnaire (P-QoL). Also included is a questionnaire that measures the expectations of self-efficacy and results in women with pelvic floor disorders after performing exercises focused on this [27]: the Broome Pelvic Muscle Self- Efficacy Scale.

Table 1 details the structural characteristics of the questionnaires. This table has been structured in seven columns: acronym, number of items, population, purpose, dimensions, subscale and punctuation. The number of items in the questionnaires varies from 6 from the UDI-6 [26] to 53 items on the EPIQ [14]. All the included questionnaires were focused only on women. The scoring scales vary greatly between the included studies, with questionnaires that are scored from 0–100 (for example, PFDI-20 [8]), being more intuitive and others from 0–10 (ex., EPIQ [14]).

Table 2 shows the psychometric characteristics of the identified questionnaires. All the questionnaires perform an analysis of internal consistency as well as test–retest reliability, with the exception of the ICIQ-SF, B-SAQ and BPMSES in this last point. From the 14 questionnaires, 2.8 of them performed a factor analysis to identify different components of the questionnaire. However, only five of them perform Kaiser–Meyer–Olkin analysis, with values ranging between 0.721 and 0.921. It is important to highlight that the construct validity is made by 14 questionnaires; however, the standardized error of the measurement is the least studied psychometric variable in all the included questionnaires; specifically, only four studies carry out the assessment of this important variable psychometric.

Table 3 shows the conclusions of all the studies analyzed. All of them conclude that the translated and validated version in Spanish of each questionnaire meets the reliability and validity requirements, and that they can be used in research and clinical practice.

## 4. Discussion

The objective of this systematic review was to carry out a synthesis analysis of validated psychometric questionnaires and with cross-cultural adaptation to Spanish for the evaluation of the different or more prevalent PFD, to collect the structural and psychometric characteristics of all the questionnaires and compare them to identify the most relevant ones for use in clinical practice. Once the identification and analysis of the psychometric characteristics of all the questionnaires had been carried out, a total of 15 questionnaires were identified that were used in these studies, focused on the different pathologies of pelvic floor. Within the questionnaires focused on PFD, QoL, sexual function, the impact of POP and urine and stool losses, pain and disability were evaluated.

Most of these questionnaires developed to assess QoL, pain, sexual function and social impact are in English, and are used both in the clinical setting and in research on Anglo-Saxon culture. There are more countries and cultures where clinical practice and research environments use the same assessment and diagnostic tools, for example Spain.

The fact that most of the questionnaires are in English is the reason that they cannot be used in all countries, since there is the problem of cultural and linguistic differences, which can pose difficulties in terms of equivalence of the questionnaires translated from the original versions. Therefore, the validation process in the desired language must be as rigorous as possible and meet homogeneous standards. This process has to allow that the diverse versions that are going to be realized and developed in different parts of the world can be adapted culturally and linguistically, in addition to being comparable with each other so that they can be used in higher evaluations, such as reviews and meta-analyses.

### 4.1. Selection and Use of Questionnaires in a Clinical and Research Setting

Four questionnaires have been identified that assess QoL [8,14,26,30], five of them measure the impact of PFD on sexual function [15,25,28,29,32], seven evaluate the symptoms of POP and UI [8,14,25,26,27,30,31], and one was focused on pregnant women [26].

All questionnaires have a number of different psychometric characteristics, as well as a different outcome variable. That is why, depending on the variable that is most interesting, one questionnaire or another will be used, and it will be the clinician who makes this decision depending on which one best suits the needs or objectives, time available, patient profile, main variable of interest, etc.

To evaluate the QoL of people suffering from PFD, four questionnaires were found [8,14,26,30], as already mentioned above. These questionnaires, despite evaluating the QoL, do not contemplate the area of sexual function and do not contain questions that deal with it; therefore, it is necessary to have other tools that do take it into account. For example, the EPIQ [14] contains 53 items and each item has a range from 0 to 10. The P-QoL [30] contains 20 items and the scale ranges from 0 to 100, the IIQ-7 [26] contains 7 items and the scale ranges from 0 to 3, and finally, the PFDI-20 [8] contains 20 items distributed in 3 blocks with a maximum score of 300.

Sexual function can be assessed with the five questionnaires that have been found in the studies (PISQ-12 [25], PISQ-IR [28], FSFI-postmenopausal [29], FSFI [15], SSS-W-E [32]). The main differences between the PISQ-IR and the PISQ-12 are found in the PISQ-IR difference between sexually active and non-active women [28], but both are focused on sexual function within pelvic floor dysfunctions. The PISQ-IR [28] is a review carried out by the IUGA of the two previous questionnaires and consists of 20 items. The most widely used questionnaire to assess sexual function in women is the FSFI-postmenopausal [29] and FSFI [15], which consists of 19 items that measure sexual dysfunction in six domains according to the woman’s perception (desire, arousal, lubrication, orgasm, satisfaction and pain) and is considered the “gold standard” to evaluate sexual function in women. The main difference between these two questionnaires is that one of them has been validated in postmenopausal women [29].

If the purpose is to evaluate POP, we find the P-QoL [30] with 20 items and the EPIQ [14] with 53, and the PIKQ [31] with 24 items. The P-QoL and EPIQ arises from the need to have a tool that evaluates the QoL specifically in women with prolapse, since the PIKQ focuses on the woman’s own knowledge of her prolapse. According to Sánchez-Sánchez et al. [30], the P-QoL assesses the severity of symptoms and the impact on QoL, in addition to serving as a guide to choose the most appropriate treatment in each case, being surgical or conservative and also offering the option of evaluating its effectiveness. 

The pathology for which most questionnaires were found was UI, with a total of eight: ICIQ-SF [23], B-SAQ [24], PISQ-12 [25], EPIQ [14], UDI-6 [26], IIQ-7 [26], Broome [27] and PIKQ [31]. In addition to evaluating the UI, it also has sections where it evaluates general data and gynecological history, general health data, overactive bladder syndrome, questions about QoL affection, genital prolapse, anal incontinence, pain and emptying difficulty, defecatory dysfunction, sexual life and sociodemographic data. Additionally, on the other hand, the PIKQ focuses on the woman’s own knowledge about UI.

Lastly, the Broome Pelvic Muscle Self-Efficacy Scale [27], as its name indicates, measures self-efficacy in performing pelvic floor exercises and takes into account the dimensions of self-efficacy and outcome expectations.

### 4.2. Psychometric Characteristics of the Questionnaires That Assess QoL

The four questionnaires [8, 14, 26, 30] identified for the evaluation of the QoL in women with pelvic floor problems had reliability values that ranged from 0.64 [8] to 0.95 [26]. This psychometric variable was in line with other versions of the same questionnaire. For example, in the study by Sánchez-Sánchez et al. [4], the PFIQ-7 and the PFDI-20 have a test–retest reliability very similar to that of the versions in other languages, such as Chinese, Swedish or Turkish [34,35,36], and we could also confirm a very good reliability, from 0.89 to 0.99, in African languages [37].

When analyzing the construct validity, Sánchez-Sánchez et al. [8] obtained a good relationship between the SF-12 Health Survey and the Spanish versions of the PFDI-20 and PFIQ-7. When comparing the PFDI-20 and PFIQ-7 with the EPIQ [14] and ICIQ-SF [23], the scores for the dimensions that measured the same symptoms showed a high correlation. Regarding internal consistency, Cronbach’s alpha ranges between 0.837 for the PFDI-20 and 0.96 for the EPIQ. Regarding construct validity, only the EPIQ did not include external validity, while the others were compared with more than one gold standard. In relation to other adapted and validated versions of the PFDI-20 and PFIQ-7, it can be observed that similar results are obtained in relation to internal consistency, such as, for example, in African languages [37], for the PFDI-20 (0.71–0.89) and the PFIQ-7 (0.81–0.89). On the other hand, the original versions of the PFDI and PFIQ [38] showed very good internal consistency with alpha values of 0.88 and 0.97, respectively. This agreement was confirmed with the development of the short form versions of these questionnaires.

### 4.3. Psychometric Characteristics of the Questionnaires That Assess Sexual Function

There are five validated questionnaires in Spanish for the evaluation of sexual function [15,25,28,29,32], as already mentioned. In three of the studies the test–retest reliability was calculated, for example FSFI [15] with an ICC value of 0.96, and PISQ-12 [25] with an ICC value between 0.22 and 0.76. On the other hand, the ICC values of the Spanish FSFI-postmenopausal [29] were substantial-to-excellent for the FSFI total score, with ICC values ranging between 0.884 and 0.972, which are comparable with those described by Takahashi et al. [39] in the analysis of the Japanese version of FSFI. In the case of the PISQ-12 [25], the best ICC value between the Spanish and English versions had a value of 0.76, showing that there was equivalence between both versions.

The internal consistency was calculated for the questionnaires related to sexual function, where Cronbach’s alpha oscillates between 0.79 of the PISQ-IR [28] and 0.964 of the FSFI-postmenopausal [29]. However, in the German version of the PISQ-IR, Cronbach’s alpha coefficients ranged from 0.64 to 0.94 [40]. On the other hand, in the original FSFI validation, Rosen et al. [40] described a high degree of internal consistency, with Cronbach’s alpha values of 0.89 and higher, and 0.97 for the total FSFI score. In addition, a validation of the Persian version [41] reported a Cronbach’s alpha greater than 0.80 for the entire scale and its dimensions, and at the same time in the Italian version [42] Cronbach’s alpha coefficients for total and domain score were sufficiently high, ranging from 0.92 to 0.97 for the total sample.

The construct validity was calculated with a gold standard in the PISQ-12, the PISQ-IR and FSFI-postmenopausal questionnaires. However, regarding the validity of the convergent construct, it was only calculated in the FSFI, where the highest correlations found were between arousal and desire, orgasm and satisfaction, and between orgasm and satisfaction, coinciding with the result of the original study. In the case of the PISQ-IR [28], factor analysis showed that the Spanish version had a similar structure to the English one.

### 4.4. Psychometric Characteristics of the Questionnaires That Evaluate POP

The test–retest reliability of the three questionnaires that evaluate the POP is high, being between 0.49 and 0.91 in the case of the EPIQ [14], 0.791 in the P-QoL [30] and 0.977 in the PIKQ [31]. However, in the case of the P-QoL questionnaire in relation to other versions [43,44,45,46,47,48,49,50,51,52], the majority of studies scored poorly for test–retest reliability largely due to the small sample sizes, poor description of test conditions, and poor stability of the retest sample.

Internal consistency was also calculated in the three instruments where Cronbach’s alpha coefficient had a value of 0.94 in the EPIQ [14] compared to 0.91 obtained in the original version [53]. On the other hand, in the PIKQ [31], Cronbach’s alpha coefficients were 0.745 and 0.758 for the UI and POP dimensions, respectively, and to the same degree as the original version [54] (0.825 and 0.895, respectively) and the Turkish version [55] (0.678 and 0.756, respectively). This shows that when all the elements measure the same construct, the intercorrelations between items increase. This coefficient was also calculated for seven dimensions identified in the EPIQ (QoL, overactive bladder, fecal incontinence, pain and difficulty emptying, defecatory dysfunction, stress urinary incontinence and POP), and in some cases they were higher than those obtained in the original version. In the P-QoL [30], this coefficient obtained high values of internal consistency and ranged between 0.550-0.877, with the exception of the dimensions “sleep/energy” and “severity measures”, which were acceptable (0.621 and 0.550, respectively). In addition, in other versions of the P-QoL analyzed, the scores were excellent [43,48,49,50,56] and good [47,51,57] scores were found in some other versions, but in most studies, the questionnaire displayed adequate evidence with Cronbach’s alpha values ≥ 0.7.

The construct validity was analyzed in the study carried out by Sánchez-Sánchez et al. [30] and they verified that there were high values and, therefore, a good relationship for the P-QoL dimension and the PFDI-20 and PFIQ-7 questionnaires. For the evaluation of this psychometric characteristic, Espuña-Pons et al. [14] carried out a factor analysis of the seven previous dimensions, which were also identified in the original version of the questionnaire: QoL, fecal incontinence, pain and difficulty emptying, defecatory dysfunction, stress urinary incontinence and POP.

### 4.5. Psychometric Characteristics of the Questionnaires That Evaluate UI

Of the eight questionnaires identified for the evaluation of UI (ICIQ-SF, B-SAQ, PISQ-12, EPIQ, UDI-6, IIQ-7, Broome and PIKQ [14,23,24,25,26,27,31]), only the value taken by the ICC is available in five questionnaires [14,25,26,32], oscillating between 0.22 [25] and 0.99 [32].

Internal consistency was also calculated in all the instruments where Cronbach’s alpha coefficient took a value between 0.667 and 0.94. In the specific case of the EPIQ questionnaire, this coefficient took the value 0.94 for the total of the items compared to 0.91 obtained in the original version [53]. At the same time, for the ICIQ-SF questionnaire, Cronbach’s alpha was calculated for the three items of the questionnaire (“frequency”, “quantity” and “affectation”), which was 0.89, resulting in being high and being practically similar to the Portuguese version [58], with a Cronbach alpha of 0.88. It is also worth noting, in the Broome questionnaire, that the Cronbach alpha coefficient was extremely high (0.91) and very close to that of the original version (0.997) [59].

### 4.6. Responsiveness

Responsiveness or sensitivity to change was measured in only five questionnaires [15,30,31,33]. Responsiveness is the ability to detect changes that occur as a result of therapy or disease progression and has been suggested as one criterion to choose among the scales used to evaluate the efficacy of a therapeutic intervention. In the case of PFDI-20, PFIQ-7 and P-QoL [30,33], were assessed with pre- and post-physiotherapy, implying that PFDI-20, PFIQ-7 and P-QoL [30,33] responsiveness can be detected to assess changing quality of life with a small change in score after physiotherapy. On the other hand, the UI and POP sections of the Spanish version of the PIKQ [31] show an excellent response capacity in women after a physiotherapy treatment that included pelvic health education interventions.

### 4.7. Limitations

It should be noted that some limitations were observed in the tools analyzed. Many of them do not have psychometric variables as important as sensitivity or error measures. Therefore, in the future it is convenient to design studies that analyze these psychometric variables, which are of great importance in research, especially in the clinical setting.

On the other hand, it is very important to take into account that native Spanish is a language spoken by more than 489 million people in the world, being the second most widely spoken language after Mandarin Chinese [18]. This group of people are in different countries. For this reason, it is essential to consider the cultural characteristics of each population group that could condition the interpretation of the questions and the answers obtained. In this sense, if the sociodemographic and cultural differences are substantial, it would be necessary to develop a specific version, fully adapted to the population group of interest. This last point was not a limitation in the present study, since these sociodemographic and cultural differences were taken into account, but it is considered important to highlight it, since many of the studies used consider it that way.

## 5. Conclusions

The main conclusion that can be drawn from this study is that the Spanish versions of the questionnaires show good basic structural and psychometric characteristics for the evaluation of patients with PFD and pathologies. Spanish clinicians have different instruments with psychometric characteristics that, as a general rule, resemble other versions of the same questionnaire published in other languages.

Therefore, these characteristics would allow the results obtained to be compared with samples from other countries. Despite these good characteristics, there are psychometric variables that none of the questionnaires selected in this study include. Therefore, it is necessary to design studies that include psychometric variables so that the validation process is homogeneous and identical for the scientific community. With regard to the responsiveness, only five questionnaires included this relevant analysis. The relevance of responsiveness lies in assessing the changes related to therapeutic interventions and must be considered in these kinds of questionnaires in future studies.

Thanks to this study it can be concluded that the psychometric questionnaires for the evaluation of pelvic floor available so far, and that have been included in this study, that are translated and validated into Spanish are those that assess QoL, the impact of PFD on sexual function, the symptoms and impact of POP and UI, and, finally, that also assess self-efficacy and outcome expectations in women with PFD after performing exercises focused on this.

## Figures and Tables

**Table 1:** Structural characteristics of the questionnaires.

Questionnaire	Acronym	Nº Items	Population	Purpose	Dimensions	Subscale	Punctuation
**International Consultation on Incontinence Questionnaire-Short Form** [23]	ICIQ-SF	3 + 8	500 women 59.4 ± 13.2 years (Mean ± SD)	Detect UI in any healthcare setting	-	-	Sum of the first 3 items (from 0 to 21 points)
**Bladder control Self-Assessment Questionnaire** [24]	B-SAQ	8	133 women 57.5 ± 15.5 years (Mean ± SD)	Identify overactive bladder problems	-	2: Discomfort and symptoms	Likert scale: from 0 to 3. The scores of each subscale are added separately
**Pelvic Organ Prolapse/ Urinary Incontinence SexualQuestionnaire** [25]	PISQ-12	12	49 women 47.9 ± 9.8 years (Mean ± SD)	Assess sexual function in women with prolapse genital and/or urinary incontinence.	-	-	Sum of the scores for each item, (from 0 = always to 4 = never), reversing this score for items 1, 2, 3, and 4.
**Epidemiology of Prolapse and Incontinence Questionnaire** [14]	EPIQ	53 (22 scoring)	120 women	Evaluates the symptoms of POP and UI, and the QoL in women who suffer from them	7: Overactive bladder, SUI, QoL, POP, FI, pain and difficulty in emptying and defecatory dysfunction	-	Each item from 0 to 10
**Urogenital Distress Inventory** [26]	UDI-6	6	181 pregnant women 33 years (Median)	Measure the parameters presence, severity, symptoms associated urogenital and type of urinary incontinence.	-	3: Irritative symptoms, stress symptoms and symptoms of obstruction/pain related to urination	Likert scale: From 0 to 4
**Incontinence Impact Questionnaire** [26]	IIQ-7	7	181 pregnant women 33 years (Median)	Measure the QoL in women with urinary incontinence	Seven components: ability to do household chores, physical activity, recreational activity, ability to travel, social activities, emotional state, and frustration	-	Likert scale: From 0 to 3
**Pelvic Floor Distress Inventory Short Form** [8]	PFDI-20	20	114 women 56 ± 11 years (Mean ± SD)	Measures QoL with health or perceived health in people with PFD and symptom scale	3: POPDI, CRADI, UDI	-	POPDI: 0–100 CRADI: 0–100 UDI: 0–100 Maximum score 300.
**Pelvic Floor Impact Questionnaire Short Form** [8]	PFIQ-7	21, 7 items for each dimension	114 women 56 ± 11 years (Mean ± SD)	Measures the impact of PFD	3: UIQ, CRAIQ, POPIQ	-	UIQ: 0–100 CRAIQ:0–100 POPIQ:0–100 Maximun score 300.
**Broome Pelvic Muscle Self- Efficacy Scale** [27]	-	23	119 women 53.5 ± 19.8 years (Mean ± SD)	Measures the expectations of self-efficacy and results perceived by women with UI who perform pelvic floor exercises	2: Expectation of self-efficacy, expectation of result	-	Each item 0–100
**Pelvic Organ Prolapse/ Urinary Incontinence Sexual Questionnaire** [28]	PISQ-IR	20	268 women: 118 NSA 64.9 ± 10.8 years 150 SA 54.7 ± 10 years (Mean ± SD)	Assess female sexual function in women with pelvic floor disorders (sexually active and inactive women)	-	2: NSA, SA	NR
**Female Sexual Function Index** [29]	FSFI	19	152 postmenopausal women 63.91 ± 6.99 years (Mean ± SD)	Assess the key dimensions of sexual function in women	-	6: Sexual desire, arousal, lubrication, orgasm, satisfaction and pain.	The subscales range from 0 (or 1) to 5 (higher scores indicate better sexual function).
**Female Sexual Function Index** [15]	FSFI	19	323 women: 167 FSD group 48 ± 8 years 156 control group 40 ± 11 years (Mean ± SD)	Evaluates sexual health in the female population	-	6: Sexual desire, arousal, lubrication, orgasm, satisfaction and pain.	The subscales range from 0 (or 1) to 5 (higher scores indicate better sexual function).
**Prolapse Quality of Life Questionnaire** [30]	P-QoL	20	200 women: 100 symptomatic 52 ± 13.4 years 100 asymptomatic 40 ± 11.1 years	Evaluates the impact of POP on the QoL of women	-	9: General health, POP impact, role limitation, physical limitation, social limitation, personal relationships, emotions, sleep/energy, severity measures	Each subscale from 0 to 100
**Prolapse and Incontinence Knowledge Questionnaire** [31]	PIKQ	24	147 women 38.57 ± 8.21 years (Mean ± SD)	Assess women’s knowledge of UI and POP	2: UI and POP sections		Score range: 0 to 1. In each scale, the minimum score is 0 and the maximum is 12.
**Sexual Satisfaction Scale for Women (SSS-W-E)** [32]	SSS-W-E	30	316 women	Measure sexual satisfaction of women	5: Satisfaction, communication, compatibility, concern for the relationship and personal concerns.		Likert scale: From 1 = strongly disagree to 5 = strongly agree.

NR: Not Reported; SUI: Stress Urinary Incontinence; UUI: Urgency Urinary Incontinence; NSA: Non-Sexually Active women; SA: Sexually Active women; PFD: Pelvic Floor Dysfunction; POP: Pelvic Organ Prolapse; UDI: Urogenital Distress Inventory; POPDI: Pelvic Organ Prolapse Distress Inventory; CRADI: Colorectal–Anal Distress Inventory; UIQ: Urinary Impact Questionnaire; POPIQ: Pelvic Organ Prolapse Impact Questionnaire; CRAIQ: Colorectal–Anal Impact Questionnaire; QoL: Quality of Life; FI: Fecal Incontinence; FSD: Female Sexual Dysfunction; UI: Urinary Incontinence.

**Table 2:** Psychometric characteristics of the questionnaires.

Study	Questionnaire	Test–Retest Reliability	Internal Consistency	Content Validity	KMO	Construct Validity	SRM
**Espuña-Pons et al., 2004** [23]	ICIQ-SF	-	Cronbach’s α = 0.89	-		-	NR
**Espuña-Pons et al., 2006** [24]	B-SAQ	-	Cronbach’s α symptoms = 0.722 Cronbach’s α discomfort = 0.889	-		ICIQ-UI SF: 0.65	NR
**Espuña-Pons et al., 2008** [25]	PISQ-12	ICC: 0.22–0.76	Cronbach’s α = 0.829	Three Factors	-	FSM: r = 0.71 FMS_DR = 0.504 ICIQ-UI-SF: r = −0.038 CACV_Simptoms_: r = −0.30 CACV_annoyance_: r = −0.40	NR
**Espuña-Pons et al., 2009** [14]	EPIQ	ICC = 0.49–0.91	Cronbach’s α Total: 0.94 QoL: 0.96 Overactive bladder: 0.91 Anal incontinence: 0.63 Pain and Difficulty emptying: 0.72 Defecatory dysfunction: 0.75 Stress Urinary Incontinence: 0.61 Pelvic prolapse = Not Calculated	Seven Factors: QoL Overactive bladder Anal incontinence Pain and Difficulty emptying Defecatory dysfunction Stress Urinary Incontinence Pelvic prolapse	-	-	NR
**Ruiz de Viñaspre et al., 2011** [26]	UDI-6	ICC = 0.812–0.902	Cronbach’s α = 0.667	-		ICIQ: r = 0.497	NR
	IIQ-7	ICC = 0.954	Cronbach’s α = 0.910			ICIQ: r = 0.472	NR
**Sánchez-Sánchez et al.,** **2013** [8] **; Sánchez-Sánchez, B. et al., 2015** [33]	PFDI-20	ICC= TOTAL= 0.644 POPDI = 0.711 CRADI = 0.771 UDI = 0.428	Cronbach’s α TOTAL= 0.837 POPDI = 0.787 CRADI = 0.630 UDI = 0.699	-		PFIQ-7: r = 0.220–0.468 PFIQ-7(UIQ): r = 0.181–0.489 PFIQ-7(CRAIQ): r = 0.212–0.397 PFIQ-7(POPIQ): r = 0.222–0.453 SF-12 (PCS) r = 0.215–0.415 SF-12 (MCS) r = 0.010–0.188 ICIQ-SF: r = 0.207–0.589 EPIQ (US) r = 0.346–0.625 EPIC (POP) r = 0.264–0.641 EPIC (QoL) r = 0.249–0.594 EPIC (CRS) r = 0.112–0.449	Total: 084 POPDI: 0.78 CRADI: 0.50 UDI: 0.67
	PFIQ-7	ICC = 0.786	Cronbach’s α ≥ 0.967	-		PFDI-20 r = 0.220–0.468 PFDI-20 (POPDI): r = 0.212–0.453 PFDI-20 (CRADI): r = 0.181–0.397 PFDI-20 (UDI): r = 0.343–0.489 SF-12 (PCS) r = 0.293–0.480 SF-12 (MCS) r = 0.173–0.352 ICIQ-SF: r = 0.427–0.567 EPIQ (US) r = 0.427–0.567 EPIC (POP) r = 0.219–0.451 EPIC (QoL) r = 0.456–0.694 EPIC (CRS) r = 0.005–0.314	Total: 0.57 UIQ: 0.61 CRAIQ: 0.39 POPIQ: 0.47
**Medrano-Sánchezet al.,** **2013** [27]	Broome Pelvic Muscle Self- Efficacy Scale (BPMSES)	NO	Cronbach’s α BPMSES = 0.91 Expectations of self-efficacy) = 0.84 Expectations of results = 0.94	Six Factors	0.721	-	NR
**Mestre et al., 2017** [28]	PISQ-IR	NO	Cronbach’s α NSA = 0.79 SA = 0.91 Subscales = 0.54–0.91	4 factors NSA condition-specific NSA partner-related NSA global quality NSA condition impact	-	POPQ: r = 0.012–0.145 Pelvic Floor Tone: r = 0.084–0.11 Oxford Scale: r = 0.01–0.141 ISI: r = 0.158–0.346 PFDI-20: r = 0.167–0.363 EPIC: r = 0.014–0.241 FSFIdesire: r = 0.528–0.871 FSFIarousal: r = 0.363–0.742 FSFIlubrication: r = 0.552 FSFIorgasm: r = 0.694 FSFIsatisfaction: r = 0.334–0.785 FSFIpain: r = 0.028–0.416 FSFItotal: r = 0.271–0.809	NR
**Pérez-Herrezuelo et al., 2019** [29]	FSFIpostmenopausal	ICC Total: 0.901 Dimension 1: 0.791–0.936 Dimension 2: 0.839–0.930 Dimension 3: 0.817–0.844	Cronbach’s α Total: 0.964 Dimension 1: 0.961 Dimension 2: 0.911 Dimension 3: 0.679	Three factors	0.921	VAS: r = 0.556–0.868 HADSanxiety: r = 0.007–0.060 HADSdepression: r = 0.004–0.061	NR
**Sánchez-Sánchez et al., 2020** [15]	FSFI	ICC FSFI total = 0.96 FSFI desire = 0.943 FSFI arousal = 0.907 FSFI lubrication = 0.939 FSFI orgasm = 0.916 FSFI satisfaction = 0.931 FSFI pain = 0.930	Cronbach’s α FSFI total= 0.850 FSFI desire = 0.760 FSFI arousal = 0.745 FSFI lubrication= 0.756 FSFI orgasm = 0.753 FSFI satisfaction = 0.753 FSFI pain = 0.765	Six Factors	0.861	-	Desire: 0.58 Arousal: 0.82 Lubrication: 0.85 Orgasm: 0.84 Satisfaction: 0.82 Pain: 1.01 Total FSFI: 1.11 In all cases *p* < 0.001
**Sánchez-Sánchez et al., 2020** [30]	P-QoL	ICC General Health Perceptions: 0.791 Prolapse Impact: 0.862 Role Limitations: 0.863 Physical Limitations: 0.908 Social Limitations: 0.864 Personal Limitations: 0.725 Emotions: 0.849 Sleep/Energy: 0.938 Severity Measures: 0.857	Cronbach’s α Role Limitations: 0.848 Physical Limitations: 0.768 Social Limitations: 0.751 Personal Limitations: 0.806 Emotions: 0.877 Sleep/Energy: 0.621 Severity Measures: 0.550	-		PFIQ-7 UIQ: r = 0.316–0.505 CRAIQ: r = 0.204–0.411 POPIQ: r = 0.305–0.555 PFDI-20 Total: r = 0.265–0.468 POPDI: r = 0.266–0.444 CRADI: r = 0.131–0.396 UDI: r = 0.235–0.513	General health: 0.29 POP impact: 0.73 * Role limitation: 0.50 Physical limitation: 0.62 * Social limitation: 0.61 * Personal relationships: 0.57 * Emotions: 0.63 * Sleep/energy: 0.65 * Severity measures: 0.73 * (* *p* < 0.001)
**Sánchez-Sánchez et al., 2021** [31]	PIKQ	ICC: PIKQ-IU: 0.995 PIKQ-POP: 0.977	Cronbach’s α PIKQ-IU: 0.745 PIKQ-POP: 0.758	-		-	PIKQ-IU: 1.16 (1.01–1.32) PIKQ-POP: 1.15 (0.99–1.33) (95%CI)
**Ruiz de Viñaspre-Hernández et al., 2021** [32]	SSS-W-E	-	Cronbach’s α Total = 0.93 Contentment = 0.86 Communication = 0.70 Compatibility = 0.81 Relational Concern = 0.90 Personal Concern = 0.93	Five Factors	Total: 0.92 Dimensions: 0.76 – 0.88	-	NR

AI: Anal Incontinence; DD: Defecatory Dysfunction; EPIQ: Epidemiology of Prolapse and Incontinence Questionnaire; FSD: Female Sexual Dysfunction; FSFI: Female Sexual Function Index; ICC: Intraclass Correlation Coefficient; ICIQ-SF: International Consultation on Incontinence Questionnaire Short-Form; KMO: Kaiser Meyer Olkin NSA: Non-Sexually Active women; OB: Overactive Bladder; PDE: Pain and Difficulty Emptying; PFDI-20: Pelvic Floor Distress Inventory Short Form; PFIQ-7: Pelvic Floor Impact Questionnaire Short Form; P; ISQ-IR: Pelvic Organ Prolapse/Urinary Incontinence Sexual Questionnaire IUGA—Revised; POP: Pelvic Organ Prolapse; QoL: Quality of Life; SA: Sexually Active women; SF-12: Quality of Life Questionnaire Short Form 12; SUI: Stress Urinary Incontinence; SRM: Standardized Response Mean; CI: Confidence Interval; NR: Not Reported; * *p* < 0.001.

**Table 3:** General conclusions of the studies.

Study	Conclusions
**Espuña-Pons et al., 2004** [23]	High sensitivity values and positive predictive values are indicators of the quality of the questionnaire as an instrument diagnosis of UI.
**Espuña-Pons et al., 2006** [24]	This questionnaire will be very useful both in clinical practice and in research, allowing epidemiological studies of the prevalence of disease evaluated from the point of view of the patient to be carried out.
**Espuña-Pons et al., 2008** [25]	The Spanish version of the PISQ-12 complies with the psychometric properties of feasibility, validity and reliability, to be used in our country, both in clinical practice and in research.
**Espuña-Pons et al., 2009** [14]	The Spanish version of the EPIQ is feasible, valid and reliable to be used in clinical practice as a screening instrument for pelvic floor pathology.
**Ruiz de Viñaspre et al., 2011** [26]	The results of the study show that both questionnaires constitute a reliable, consistent and valid instrument to evaluate urogenital symptoms and their impact on the QoL of pregnant women.
**Sánchez-Sánchez et al., 2013** [8] **;** **Sánchez-Sánchez, B., 2015** [33]	The Spanish versions of the PFDI-20 and PFIQ-7 are equivalent in content, semantics, conceptually and idiomatically with the original versions, in addition to being reliable, valid and feasible and responsive to evaluate the symptoms and QoL in Spanish women with PFD.
**Medrano-Sánchez et al., 2013** [27]	The Spanish version of the Broome questionnaire for self-efficacy is a useful measurement tool for a relevant psychometric and clinical estimation of women in performing pelvic floor exercises.
**Mestre et al., 2017** [28]	The Spanish version of the PISQ-IR meets the criteria of feasibility, validity and reliability for use in clinical practice.
**Pérez-Herrezuelo et al., 2019** [29]	The Spanish version of the FSFI shows good internal consistency and test–retest reliability, and also good construct, concurrent, and divergent validity for a population of postmenopausal women, shows satisfactory general psychometric properties and is able to discriminate between women with and without sexual dysfunctions among a population of Spanish postmenopausal women.
**Sánchez-Sánchez et al., 2020** [15]	The Spanish version of the FSFI can be used as a reliable, valid, responsive and feasible instrument to assess sexual function in women.
**Sánchez-Sánchez et al., 2020** [30]	The Spanish version of the P-QoL has sufficient validity, reliability, responsiveness, and feasibility for assessing the severity of symptoms and their impact on the QoL of Spanish women with POP.
**Sánchez-Sánchez et al., 2021** [31]	The Spanish PIKQ is a comprehensible, valid, reliable, feasible, and responsive-to-change tool for assessing patient knowledge about UI and POP conditions in the Spanish language, as well as the effect of educational treatment strategies on them, both in research and clinical interventions.
**Ruiz de Viñaspre-Hernández et al., 2021** [32]	The Spanish version has good overall reliability and validity. The findings are largely compatible with the initial hypothesis, which make the SSS-W-E a useful tool for the evaluation of women’s sexual satisfaction in clinical practice and research, in Spain.

## Data Availability

Data is contained within the article.
